# Biomechanical comparison of radiopalmar double plating with conventional palmar plating in comminuted distal radius fractures

**DOI:** 10.1007/s00402-026-06224-4

**Published:** 2026-02-24

**Authors:** Conrad-Friedrich Jäger, Christian Spiegel, Felix Christian Kohler, Heike Kielstein, Ivan Zderic, Boyko Gueorguiev-Rüegg, Gunther Olaf Hofmann, Mark Lenz, Wolfram Weschenfelder

**Affiliations:** 1 Department of Trauma, Hand and Reconstructive Surgery, Jena University Hospital, Jena, Germany; 2 Institute of Anatomy and Cell biology, University Hospital in Halle, Halle (Saale), Germany; 3 AO Research Institute Davos, Davos, Switzerland

**Keywords:** Distal radius fracture, Biomechanic, Dorsal comminution, Locking plate, Radial buttress plate

## Abstract

**Purpose:**

The aim of this study is to analyse the effect of an additional radial buttress plate for palmar plate osteosynthesis in an AO/OTA 2R3 C2.1 fracture model.

**Methods:**

Nine pairs of freshly frozen radii were analysed for pathology and bone mineral density and divided into two matched groups. One group was treated with a variable-angle palmar locking plate alone, while the second group received an additional radial buttress plate for radiopalmar double plating. An AO/OTA 2R3 C2.1 fracture was created in all specimens. The biomechanical tests were performed according to previously published protocols. Stiffness, axial displacement of the construct, as well as fragment-specific movements and rotations were assessed.

**Results:**

No implant failure was observed. In the total cohort, stiffness increased (*p* < 0.01) and axial construct displacement decreased (*p* < 0.05). The mobility of the ulnar fragment to the shaft during cyclic testing was lower with double plating, both at baseline and endpoint (all *p* < 0.01). Fragment movements increased over the course of testing and were significant for the radial articular fragment relative to the shaft in the total cohort (*p* < 0.01). Baseline rotation of the ulnar fragment and endpoint rotation of the radial fragment in relation to the shaft were lower with double plating (all *p* < 0.05). In both constructs, the rotation of the ulnar fragment relative to the shaft was lower than that of the radial fragment at both timepoints (all *p* < 0.05).

**Conclusion:**

Biomechanically, the addition of a radial buttress plate to a standard palmar locking plate did not alter global construct stiffness, but demonstrated advantages in fragment-specific stability in comminuted distal radius fractures.

## Introduction

Locking plate technology has established palmar plate osteosynthesis as the standard of care for operative treatment of distal radius fractures [1–4]. The introduction of variable-angle (VA) locking plates has further refined fragment-specific fixation, particularly in multifragmentary fracture configurations. Although favourable outcomes are achieved in most cases, complex fracture patterns with extensive comminution and compromised bone quality—especially in severe osteoporosis—remain challenging, and implant-related complications are not uncommon [5, 6]. These geriatric fractures often show bayonet and fourchette malalignment, frequently associated with metaphyseal dorsal or dorsoradial comminution. However, previous biomechanical studies have focused solely on dorsal defect zones and have not accounted for the additional instability introduced by dorsoradial bone loss [7, 8]. For fixation of complex fractures involving the radial styloid process, several authors have proposed supplementary or even isolated radial plating as a means of improving stability [9–11]. One case series even considered this in the sense of buttress plating via the Flexor carpi radialis (FCR) approach, although its biomechanical advantage has not been investigated to date [12].

In the present human cadaveric study, a distal radius fracture model AO/OTA (Association for the Study of Internal Fixation/ Orthopaedic Trauma Association) 2R3 C2.1, representing a simple sagittal articular fracture with metaphyseal comminution, was created. The metaphyseal comminution was deliberately located in the dorsoradial region as part of the experimental design. A palmar VA locking plate osteosynthesis as sole stabilisation was compared with a palmar locking plate osteosynthesis plus radial buttress plate. The null hypothesis was that double plate fixation does not achieve measurably better fixation than the standard procedure.

## Material and methods

### Implants

**Fig. 1:**
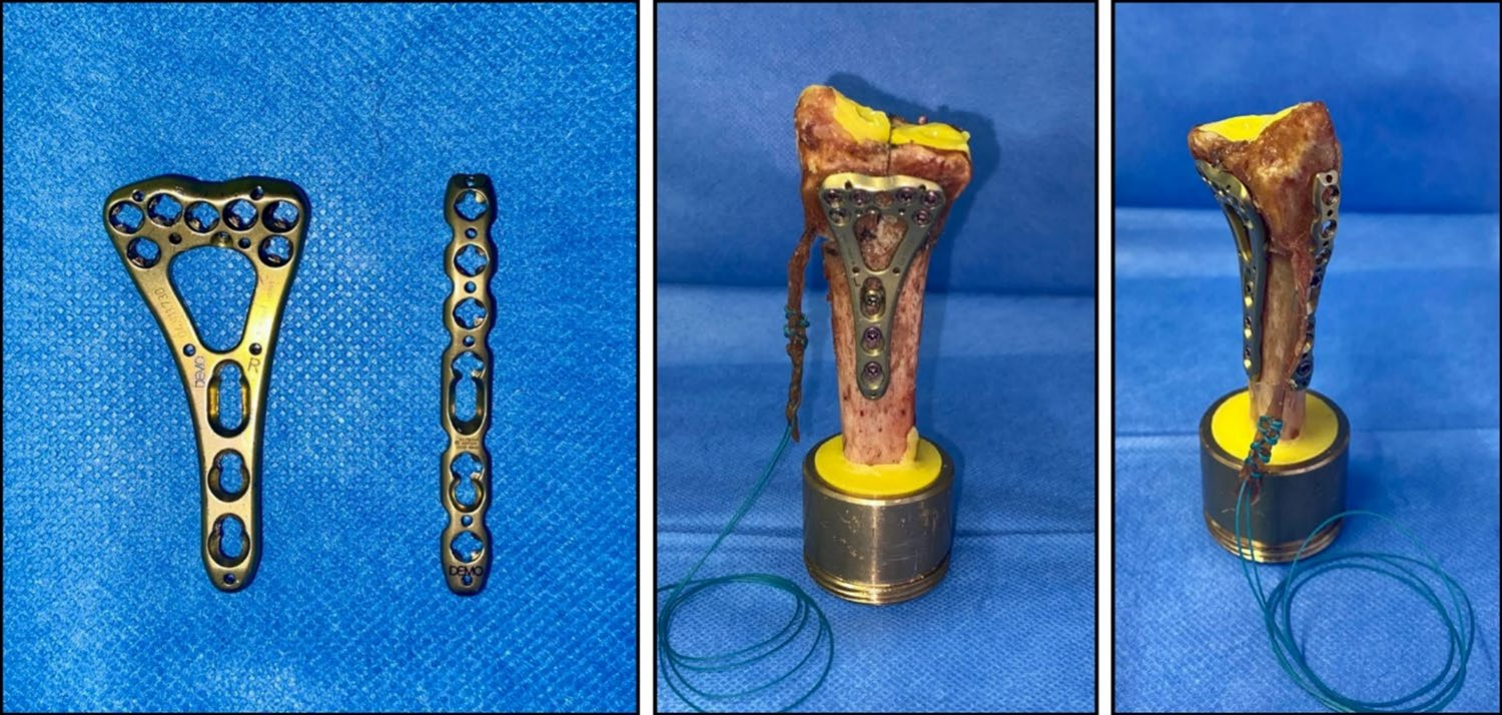
Left: Palmar 7 + 3 2.4 mm VA LCP two-column distal radius plate, 6-hole radial 2.4 mm VA LCP dorsal distal radius plate; Middle: Left distal radius with fracture, fixed with palmar 2.4 LCP, BR tendon with attached suture; Right: Right distal radius with fracture, fixed with palmar 2.4 LCP and radial 2.4 LCP, BR tendon with attached suture

Palmar plating was performed with the 7 + 3 2.4 mm VA LCP two-column distal radius plate (DePuy Synthes, Zuchwil, Switzerland). The additional radial plate was the 6-hole radial 2.4 mm VA LCP dorsal distal radius plate (DePuy Synthes, Zuchwil, Switzerland). All plates and screws are made of Ti-6Al-7Nb alloy (TAN). The implants are shown in Fig. 1.

### Bone specimens

Nine pairs of fresh-frozen radii were used. All donors had provided written informed consent for general scientific use during their lifetime. Specimens were stored at − 21 °C. Each radius was screened for osseous pathology, and bone mineral density (BMD) was quantified using quantitative computed tomography (qCT; GE Revolution EVO 64, GE Healthcare Technologies Inc., Chicago, USA). Pairs were allocated such that the right radius received combined palmar-and-radial plating, whereas the left radius underwent palmar plating only (Fig. 1). Prior to preparation and testing, the radii were thawed and all soft tissues were removed except for the brachioradialis (BR) tendon. The tendon was shortened to standardized length of 60 mm from its insertion and secured using a baseball stitch for later attachment of weight.

### Fracture model and biomechanical testing

**Fig. 2:**
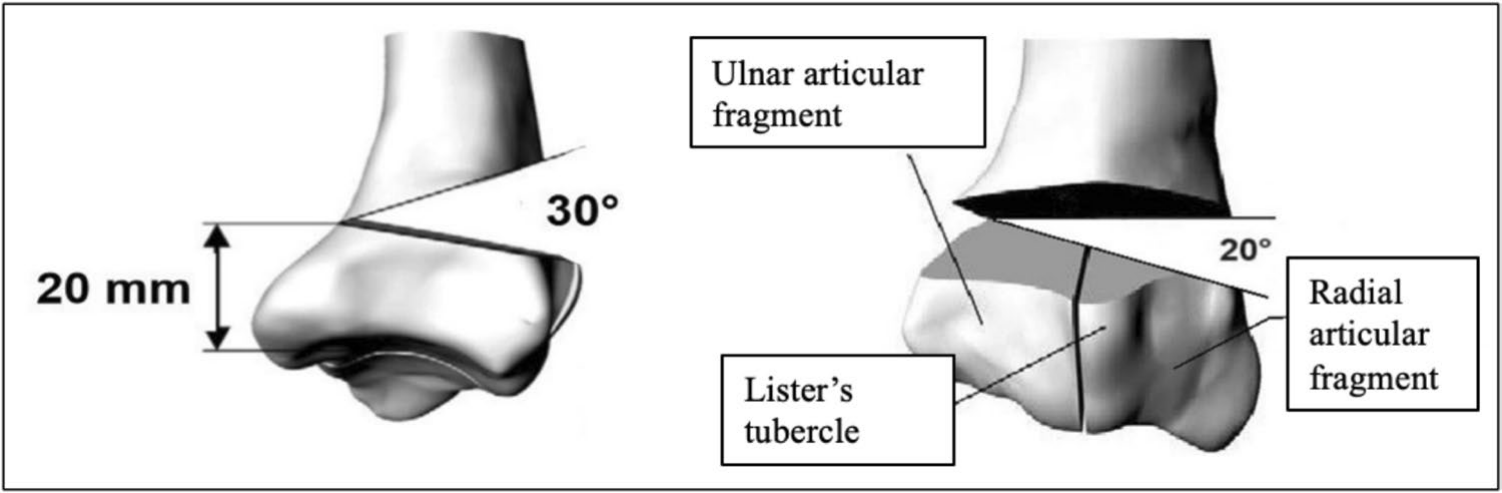
Scheme of the fracture model with a radial and dorsal zone of comminution (modified for radiodorsal zone of comminution from Schlonski [13])

First, the palmar radius plate was placed on the radii and aligned distally along the watershed line and proximally along the shaft axis. It was then fixed distally in the 3 radial and 3 ulnar screw holes with locking screws. The centre screw hole of the distal row was not occupied. The shaft was then fixed with a cortical screw, which was inserted in the oblong hole. The planned osteotomies were marked with a permanent marker and then set with a jigsaw (Bosch PMF 220 CE, Robert Bosch Power Tools GmbH, Leinfelden-Echterdingen, Germany). The aim was to simulate an AO/OTA 2R3 C2.1 fracture with a metaphyseal radiodorsal defect zone. Accordingly, a 30° radiodorsal wedge was sawed and removed 20 mm proximal to the lunate fossa. The intra-articular fracture component was then created exactly at the radial edge of the lunate fossa at the level of the middle distal screw hole (Fig. 2). The cortical screw was then loosened, the plate proximalised, and the fracture reduced—at its only contact point—the ulnopalmar angle. The cortical screw was then retightened and two further locking screws were inserted into the shaft holes. A 6-hole radial plate was then attached radially to the right radii where radiopalmar double plate osteosynthesis was to be performed after appropriate pre-bending. The plate was bent so that it acted as a buttress plate. It was then pressed against the bone proximally in the longitudinal hole with a cortical screw and the very proximal screw hole was secured with a locking screw. The remaining screw holes of the radial plate were intentionally left unfilled. This limited fixation was chosen to avoid relevant extension of the surgical exposure and to simulate distal positioning of the plate beneath the brachioradialis tendon. As the radial plate primarily serves as a buttress against fragment displacement rather than as a load-bearing implant, full screw occupancy was considered biomechanically unnecessary.

The radii were then cut to a length of 120 mm using a jigsaw. They were then aligned exactly vertically along their longitudinal axis and fixed in a conical mould, which was filled with Polymethylmethacrylate (PMMA, Technovit 3040; Heraeus Kulzer GmbH, Wehrheim, Germany). On the articular surface small metal spheres were used for the scaphoid fossa and lunate fossa embedded in PMMA to allow free rotation of the fragments. A weight of 500 g was suspended from the loop in the augmentation suture of the BR tendon via a pulley to simulate constant tension of this tendon and a constant associated radial force on the radial articular fragment.

**Fig. 3:**
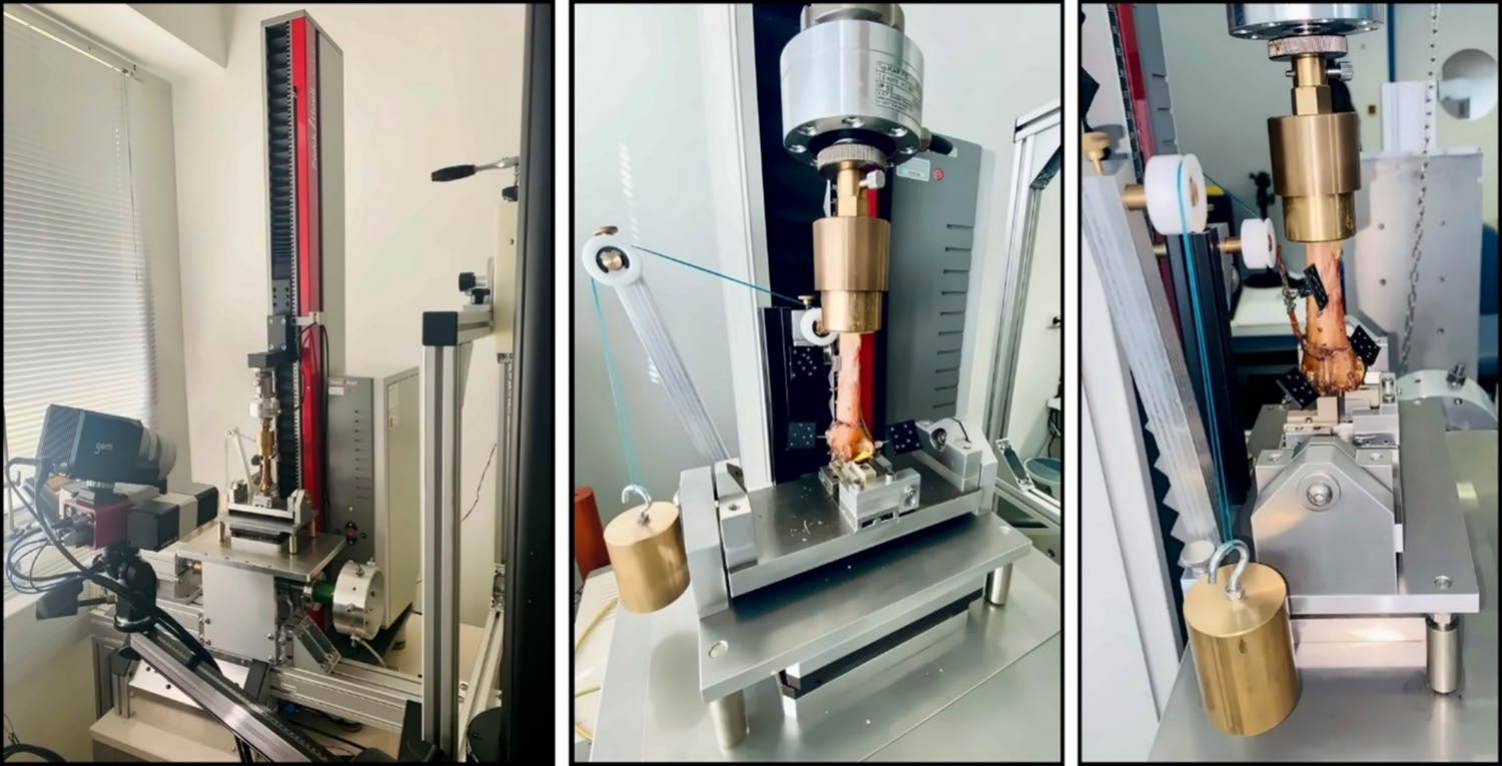
Left: Universal testing machine with GOM camera in the foreground; Middle: Test setup showing compensator and testing machine in the sagittal plane; Right: Test setup in the coronal plane

The tests were performed on a universal testing machine with a 1kN load cell (Zwick 1.0; Zwick GmbH, Ulm, Germany) in a test setup adopted from previous biomechanical studies [7, 8, 14]. Load was transferred via the shaft to a custom-made compensator ensuring a load transfer ratio of 60% through the scaphoid fossa and 40% through the lunate fossa. The compensator was also designed in a way that the scaphoid part was movable in medio-lateral alignment. This setup was mounted on a mobile x–y table to allow static determination of the mechanical system (Fig. 3).

### Data acquisition and statistical analysis

Each specimen was subjected to a three-part biomechanical testing procedure consisting of an initial quasi-static, a dynamic, and a quasi-static final test. The two quasi-static tests were identical in structure and each one consisted of three setting cycles and three measuring cycles with a peak force of 150 N and a frequency of 0.2 Hz. This allowed determination of the specimen’s stiffness and axial shortening of the construct under compressive loading, defined as the crosshead displacement of the testing machine during force application at the beginning and the end of testing. Furthermore, a camera system simultaneously recorded the movement and rotation of the individual fragments (GOM Testing controller, Carl Zeiss GOM Metrology GmbH, Braunschweig, Germany). The dynamic test consisted of sinusoidal loading with 150N peak force at a rate of 0.2 Hz over 5000 cycles.

Statistical analysis was performed using SPSS software (V.28, SPSS Inc., Chicago, IL, USA). Most variables were not normally distributed so non-parametric tests were conducted. The Mann–Whitney U-test was used to screen for differences between the groups and the Wilcoxon Signed-Rank test was applied for the comparison of metric variables within each group. The level of significance was set at 0.05.

## Results

There was no case of implant failure or loosening. Two radii, one left and one right, had to be excluded due to loosening of the PMMA embedding of the metallic spheres at the joint surface. The results of the remaining 16 radii are presented in Table 1.

**Table 1:** BMD, stiffness and axial displacement as measured with the testing machine within the two groups (n = 16)

Device	BMD (mg/cm^3^)	Initial stiffness (N/mm)	Final stiffness (N/mm)	Initial axial displacement (mm)	Final axial displacement (mm)
Palmar locking plate	184.0 (152.8–242.9)	193.7 (122.6–216.6)	195.7 (163.5–255.3)	0.78 (0.69–1.30)	0.77 (0.60–0.92)
Radiopalmar locking plate	195.2 (145.8–252.3)	207.3 (160.9–299.1)	269.5 (197.1–322.7)	0.72 (0.50–0.93)	0.56 (0.47–0.76)
*p* values	0.959	0.328	0.105	0.328	0.105

Data presented are median and Inter-Quartile-Range (IQR)

There was no significant difference in BMD between the two groups (*p* = 0.96). Although baseline and endpoint stiffness were higher in the double plating group, these differences were not significant (*p* = 0.33 and *p* = 0.11 respectively). Stiffness increased significantly during testing in both subgroups (all *p* < 0.05) and the entire cohort (*p* < 0.01). Axial construct shortening was higher in the palmar plate group at baseline and endpoint; however, this difference was not significant (*p* = 0.33 and *p* = 0.11 respectively). During testing axial construct shortening decreased. This effect was significant in the palmar plating group and the entire cohort (all *p* < 0.05), but not within the double plating group (*p* = 0.12).

**Fig. 4:**
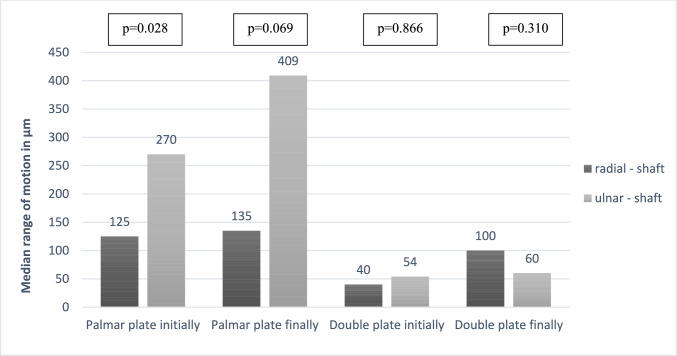
Interfragmentary displacement of the articular fragments to the shaft within both groups at the initial and final quasi-static test (n = 16)

The results regarding interfragmentary displacement between shaft and articular fragments measured by the optical camera are shown in Table 2. There were no significant differences in relative motion between the radial articular fragment and the shaft (*p* = 0.12 and *p* = 0.13 respectively) or between the two articular fragments (*p* = 0.65 and *p* = 0.23 respectively). The median interfragmentary displacement between the shaft and the ulnar articular fragment was 270 µm in the palmar plate group and 54 µm in the double plating group at baseline (*p* < 0.01). Similarly, interfragmentary displacement at the endpoint was significantly higher in the palmar plate group (409 µm) compared to the double plating group (60 µm; *p* < 0.01). In addition, the results demonstrate an increasing fragment mobility from baseline to endpoint measurements. This increase was significant in the total cohort for the relative motion between the radial articular fragment and the shaft (*p* < 0.01; ulnar-shaft *p* = 0.09; radial-ulnar *p* = 0.53). Figure 4 illustrates the interfragmentary displacement of the respective articular fragments relative to the shaft at baseline and endpoint in both groups. Interfragmentary displacement of the ulnar articular fragment relative to the shaft was significantly greater than that to the radial articular fragment within the palmar plating group (*p* < 0.05).

**Table 2:** Initial and final interfragmentary displacement within the two groups (n = 16)

Device	Initial interfragmentary displacement (µm)			Final interfragmentary displacement (µm)		
	Radial–shaft	Ulnar–shaft	Radial–ulnar	Radial–shaft	Ulnar–shaft	Radial–ulnar
Palmar locking plate	125 (73–198)	270 (189–417)	59 (33–68)	135 (120–311)	409 (138–618)	65 (36–84)
Radiopalmar locking plate	40 (38–109)	54 (20–76)	39 (20–70)	100 (39–204)	60 (32–95)	40 (30–59)
*p* values	0.121	*0.005	0.645	0.130	*0.010	0.232

Data presented are median and Inter-Quartile-Range (IQR); values for p highlighted with * and bold if p≤0.05

**Fig. 5:**
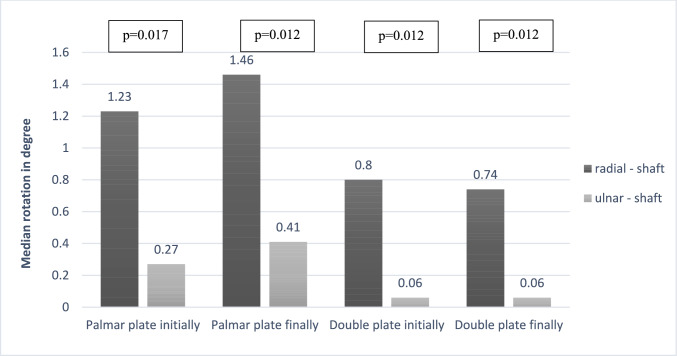
Rotation between the articular fragments and the shaft within both groups at the initial and final quasi-static test (n = 16)

The camera also recorded the rotation of the fragments in relation to each other, and these results are shown in Table 3. There was no significant difference regarding baseline rotation between radial articular fragment and shaft (p = 0.08) or between the two articular fragments (*p* = 0.65). Baseline rotation of ulnar articular fragment to shaft showed a significant difference between the two groups (*p* < 0.05). Endpoint rotational measurements showed a different distribution. No significant difference could be detected between the articular fragments and between the ulnar articular fragment and shaft (*p* = 0.51 and *p* = 0.13 respectively). The rotation of the radial joint fragment to the shaft differed significantly between the two groups (*p* < 0.05). Within the entire cohort the values of rotation show an increasing rotation of both articular fragments to the shaft and a decrease of rotation between the articular fragments. However, these trends are not statistically significant (radial–shaft *p* = 0.35; ulnar–shaft *p* = 0.06; radial–ulnar *p* = 0.14). Figure 5 showed the rotation of the respective articular fragments to the shaft at baseline and endpoint within the two groups. The rotation of the ulnar articular fragment against the shaft was significantly lower than that of the radial articular fragment in both groups at both measurement times (all *p* < 0.05).

**Table 3:** Initial and final rotation between the main fragments within the two groups (n = 16)

Device	Initial rotation (degree)			Final rotation (degree)		
	Radial–shaft	Ulnar–shaft	Radial–ulnar	Radial–shaft	Ulnar–shaft	Radial–ulnar
Palmar locking plate	1.23 (0.93–1.90)	0.27 (0.19–0.41)	0.24 (0.14–0.31)	1.46 (1.08–1.87)	0.41 (0.14–0.62)	0.18 (0.12–0.37)
Radiopalmar locking plate	0.80 (0.62–1.42)	0.06 (0.02–0.17)	0.16 (0.14–0.36	0.74 (0.49–1,49)	0.06 (0.03–0.20)	0.16 (0.10–0.21)
*p* values	0.083	*0.050	0.645	*0.050	0.130	0.505

Data presented are median and Inter-Quartile-Range (IQR); values for p highlighted with * and bold if p≤is less than or equal to≤0.05

## Discussion

Palmar locking plate osteosynthesis is currently a standard procedure for the surgical treatment of distal radius fractures and has been extensively investigated in clinical and biomechanical studies [1, 7, 8]. The test setup and cyclic loading protocol used in the present study were adopted from previously published biomechanical investigations and were designed to simulate repetitive low-load conditions during early postoperative functional rehabilitation [7–9, 14, 15]. Specifically, the number of load cycles was chosen to approximate physiological loading during the first weeks following surgery, when active wrist and finger motion is encouraged but high-load activities are avoided. Although the exact in vivo forces acting on the distal radius are not fully known, prior studies have reported compressive loads ranging from approximately 90 to 250 N during light active wrist and digit motion. Based on these data, a peak load of 150 N over 5000 cycles was selected to represent clinically relevant early functional use rather than failure loading.

Earlier biomechanical studies predominantly focused on fracture models with a dorsal metaphyseal defect. In contrast, the present study specifically reproduced a dorsoradial metaphyseal defect. While precise epidemiological data on the frequency of this fracture pattern are limited, it is commonly encountered in osteoporotic bone and is considered clinically relevant due to its association with fragment instability and loss of reduction. In addition, the present model incorporated constant tension of the radial articular fragment via the brachioradialis tendon, thereby more closely approximating physiologic loading conditions.

Martinez-Mendez et al. reported loss of reduction in 12.1% of osteoporotic patients following palmar locking plate fixation [6]. Berglund and Messer identified dorsiflexion, loss of radial length, and collapse of the lunate facet as the three predominant modes of failure in dorsally displaced fractures [16]. In cases with extensive comminution or poor bone quality, several authors have described the use of screw augmentation or combined fixation strategies to enhance stability [9, 17–20]. Although some biomechanical studies suggest greater stability with combined dorsopalmar plating in complex distal radius fractures, clinical trials have not confirmed this benefit and report higher rates of infection, hardware removal, and extensor tendon complications [21, 22]. These findings highlight the need for fixation strategies that selectively address fragment instability without substantially increasing surgical morbidity.

Jacobi et al. introduced the concept of an additive palmar buttress plate with sole proximal screw fixation for palmar locking plate osteosynthesis in distal radius fractures in 2010 [12]. In 2012, Helmerhorst published a retrospective case series describing the use of an additional radial plate via the extended FCR approach according to Orbay, reporting favourable radiological and functional outcomes in intra-articular distal radius fractures; however, in contrast to Jacobi, distal screws were also inserted [23, 24]. Only few clinical reports have addressed radial plating of the distal radius. While Jacobi and Helmerhorst used radiopalmar double plating, Wei applied radial plating alone. In these small case series, complication rates were reported to be low, with no clinically relevant irritation of the superficial radial nerve or the tendons of the first extensor compartment [12, 23, 25]. However, these studies are limited by small sample sizes and retrospective designs and may therefore be underpowered to detect infrequent but clinically relevant complications. Potential risks associated with radial plating, including irritation of the superficial radial nerve or extensor tendon pathology, should therefore be considered when interpreting these findings. Despite these encouraging clinical reports, biomechanical evidence regarding radiopalmar double plating remains inconsistent. Blythe et al. found no biomechanical advantage in extra-articular fracture models, whereas Grindel et al. reported increased stability, although their setup included an additional distal pin in the radial plate [9, 10]. This inconsistency may partly be explained by differences in fracture patterns and by the limited ability of global biomechanical parameters to detect fragment-specific instability.

In the presented study, no fixation failure occurred. Global construct stiffness and axial displacement measured via the testing machine did not differ significantly between palmar plating and radiopalmar double plating. Consistent with earlier biomechanical studies, construct stiffness increased and axial shortening decreased following cyclic loading [7, 8]. However, global construct parameters alone may not adequately reflect clinically relevant instability at the level of individual articular fragments, particularly in comminuted fracture configurations.

By contrast, the fragment-specific analysis using a high-precision optical tracking system revealed significantly reduced interfragmentary displacement of the ulnar articular fragment relative to the shaft when an additional radial plate was applied, both before and after cyclic loading. Fragment rotation relative to the shaft was likewise reduced in the double-plating group, with significant differences observed for the ulnar fragment prior to cyclic loading and for the radial fragment following cyclic loading, while the radial fragment consistently demonstrated greater rotational mobility than the ulnar fragment. The differing rotational behavior of the radial and ulnar articular fragments may be related to their distinct anatomical positions, fragment geometry, and fixation characteristics. Pre-cyclic rotational instability of the ulnar fragment may primarily reflect the initial fixation configuration and fragment support, whereas post-cyclic rotational differences of the radial fragment may be influenced by repetitive loading, construct settling, and progressive micro-motion under cyclic conditions. These interpretations remain speculative but biomechanically plausible given the asymmetric load distribution and fragment orientation in distal radius fractures.

Although the optical tracking system enabled highly precise quantification of fragment motion, the present analysis focused on the magnitude of translational and rotational motion rather than direction-specific movement patterns. Consequently, detailed assessment of the exact directions of fragment displacement or rotation was beyond the scope of this study and should be addressed in future investigations.

Taken together, these findings suggest that radiopalmar double plating primarily improves fragment-level stability rather than global construct stiffness. The additional radial buttress plate appears to stabilize the radial articular fragment relative to the ulnar fragment, thereby limiting rotational freedom and indirectly constraining motion of the ulnar fragment relative to the shaft. This mechanism may help explain why fragment-specific differences were detectable despite similar global stiffness values. As highlighted by Berglund and Messer, failure of the lunate facet represents a major mode of failure in distal radius fractures, underscoring the clinical relevance of improved fragment-specific stabilization [16].

A limiting factor in this study is that the cadaver model used represents the fracture situation in a very simplified manner; for example, relevant stabilising structures were absent due to removal of the ulna and surrounding soft tissues. A strength of this study is its relatively large sample size using human cadaver bones with a standardised fixation. Furthermore, this study is the first in which a high-precision camera system was used in addition to direct measurement with the testing machine, enabling the assessment of detailed interfragmentary motion.

## Conclusion

The present study of human cadaveric radii showed a biomechanical advantage of the additive radial buttress plate over the standard palmar locking plate in unstable C2 fractures of the distal radius involving a radiodorsal defect zone, a pattern commonly encountered in osteoporotic bone. Prospective clinical studies comparing the two fixation methods are necessary to prove the clinical benefit of additional fixation in highly unstable distal radius fractures.

## Data Availability

The data presented in this study are not publicly available but available on request from the corresponding author. The data are not publicly available due to privacy and ethical restrictions.

## Abbreviations

AO/OTA: Association for the Study of Internal Fixation/ Orthopaedic Trauma Association
FCR: Flexor carpi radialis
TAN: Titanium aluminium nitrite
BMD: Bone mineral density
qCT: Quantitative computed tomography
PMMA: Polymethylmethacrylate
IQR: Inter Quartile Range
